# Predictors of Mental Health Outcomes in Grocery Store Workers amid the COVID-19 Pandemic and Implications for Workplace Safety and Moral Injury

**DOI:** 10.3390/ijerph18168675

**Published:** 2021-08-17

**Authors:** Melissa Janson, Jill D. Sharkey, Daniel A. del Cid

**Affiliations:** Gevirtz Graduate School of Education, Department of Counseling, Clinical, and School Psychology, University of California, Santa Barbara, CA 93106, USA; jsharkey@ucsb.edu (J.D.S.); delcid@ucsb.edu (D.A.d.C.)

**Keywords:** mental health outcomes, grocery store workers, threat perception, COVID-19, moral injury

## Abstract

Limited research exists on the mental health (MH) of grocery store workers (GSWs), who have been on the frontlines throughout the COVID-19 pandemic. A disaster MH conceptual model incorporating demographics, disaster exposure and threat (COVID-19 fear and workplace threat perception), perceived stress, and social support (lack of from family and friends) was utilized to predict MH outcomes (anxiety, depression, and post-traumatic stress symptoms; PTSS) of GSWs. GSWs (*n* = 842) were recruited through a regional union in California. The participants were diverse (62.1% female) and were 18–69 years of age (*M* = 41.5, *SD* = 13.9). They completed an online survey regarding COVID-19 fear, workplace threat perception, perceived stress, lack of social support, and workplace needs/recommendations for support. Three hierarchical linear regression models were run assessing each MH outcome. Thematic analysis coding and an inductive approach were utilized for analyzing open-ended responses of workplace needs/recommendations. Females and younger GSWs (ages 18–29 years old) on average, reported higher MH symptoms than males and older age groups, respectively. COVID-19 fear and perceived stress were significant predictors of anxiety, while COVID-19 fear, workplace threat perception, and perceived stress significantly predicted depression and PTSS, explaining almost half of the variance for each model. Social support and demographics were not predictive of MH outcomes. Almost half of GSWs (40%) requested increased safety protections in the workplace. Feelings of fear of COVID-19, threat in the workplace, and overall perceived stress are predictive of GSWs’ MH outcomes. Increasing feelings of safety in the workplace and reducing stress may lessen MH symptoms.

## 1. Introduction

Grocery store workers (GSWs) have worked on the frontlines since the start of the COVID-19 pandemic and can be considered the “unexpected, but under protected… heroes” during this time [1] (p. 2006). In general, GSWs may be comprised of vulnerable individuals from low-income and racially diverse backgrounds, which may contribute to social and health inequities [2]. Essential workers such as GSWs may also be treated as easily replaceable, which may contribute to a lack of efforts being made to establish clear legal protections or safety guidelines in the workplace without repercussions [1].

This past winter, GSWs in California and throughout the U.S. faced rising COVID-19 cases. According to a news article, more than 800 Southern California grocery store and food system workers tested positive for COVID-19, with outbreaks at more than 137 Los Angeles area supermarkets in November and December 2020 [3]. There have been close to four million confirmed cases of COVID-19 and 64,328 deaths in California, as of 10 August 2021 [4]. Interacting with the public on a regular basis, GSWs have increased chances of contracting COVID-19 while working, putting their lives at risk [2]. GSWs have made headlines in past news coverage for having to engage in uncomfortable and potentially threatening interactions with customers who defy mask wearing mandates within stores [5]. Because of such experiences, working as a GSW during a global pandemic may be particularly stressful. Research is needed to understand how the threat of COVID-19 and experiences at work impact GSWs. Thus far, research on essential workers has primarily focused on healthcare workers, and although more research is needed pertaining to this population, limited studies on other essential workers exist. Little is known about the impact of COVID-19 on the mental health (MH) and psychosocial functioning of GSWs.

Essential workers, like GSWs, may be particularly vulnerable to physical and psychological health impacts during the COVID-19 pandemic as they provide critical infrastructure to communities while actively engaging with the public on a regular basis. As a result, GSWs face increased risk of exposure to COVID-19. In a single Boston grocery store, employees with direct customer interaction were five times more likely to test positive for COVID-19 than the surrounding communities, most of whom were asymptomatic [6]. Several news organizations reported stories of GSW deaths and widespread infections in the early months of the pandemic [7,8]. Significant asymptomatic infection rate and exposure risks were associated with psychological distress of GSWs [6]. The inability to engage in social distancing while at work was a significant risk factor for anxiety and depressive symptoms [6]. GSWs may also experience COVID-19 health anxiety, which is a heightened and continued state of vigilance toward one’s body for COVID-19 related-symptoms. COVID-19 health anxiety can result in cycles of increased anxiety, endorsement of a greater number of COVID-19 related symptoms, and misreading of one’s health, even amid negative COVID-19 tests [9].

Thus far, limited studies have revealed that GSWs are experiencing MH symptoms and distress. About 24% of a study sample of GSWs met criteria for at least mild anxiety or greater, while 8% met criteria for at least mild depression [6]. Results from the Arizona Frontline Worker Survey found that female frontline workers in the grocer and food processing industries reported more anxiety, depression, and stress than males, and younger workers reported more distress than older workers (55 years of age and above) [10]. Additionally, grocer and food processing workers reported distress three times greater than national average scores and majority (60%) reported above-average levels of stress [10]. High levels of grocer and food processing workers’ concerns were related to safety measures, especially those pertaining to customers and their behavior [10]. Less than a quarter of all grocer and food processing workers (22%) reported they believed it was likely they would be assaulted by a customer and over half (54%) believed it was likely they would be threatened by a customer for enforcing safety guidelines [10]. Perceptions of safety in the workplace were found to be the greatest predictor of MH [10]. The MH of GSWs may be affected by fear of contracting COVID-19, increased demands and stress at work, and whether COVID-19 workplace safety measures are enforced or not. Further, GSWs may be seen as low status due to irregular working hours and low pay in comparison to health care workers, which may further exacerbate stress reactions.

GSWs may also be vulnerable to experiencing moral injury, psychological distress which can result from actions or an absence of them, that go against one’s moral or ethical code, or of experiences of disregard or betrayal from leadership or trusted individuals [11]. Such experiences may involve leadership failing to adequately protect, advocate on behalf of, or support workers’ well-being or safety during the COVID-19 pandemic. Moral injury may contribute to negative thoughts about oneself or others [11] or MH problems like depression, PTSS, and anxiety [12]. GSWs may grapple with the difficult decision of whether to continue working, even though there are increased risks of illness and death for themselves, and their loved ones. Worries about contracting COVID-19 while at work, and possibly bringing the virus home and infecting loved ones were found to be higher for GSWs than health care workers [13]. Many GSWs may feel as though they have no choice, except to work to financially support themselves and their loved ones [2]. Possible reactions to this dilemma may include outrage, despair, grief, guilt, or remorse toward their employers, work situation, or personal decisions, which can contribute to distress [2].

Some efforts have been made by several grocer retailers to instate measures to protect their employees, such as limiting the number of customers in a store at a given time, adding plexiglass partitions at the register [1], and requiring masks to be worn within the store [10]. The impact of such measures aimed at increasing workplace safety should be documented and studied, as their impacts on GSW well-being, health, and MH functioning are not fully understood yet.

In general, disasters such as the COVID-19 pandemic can create long-term stressors that add to distress and anxiety and can exact a general toll on the quality of everyday health and wellbeing leading to long-term effects on physical, MH, and psychosocial functioning of those affected [14]. A review of risk and resilience in individuals, families, and communities after disaster found that approximately up to 30% of disaster exposed survivors may experience serious psychological and physical impairments [14]. A recent review of MH outcomes throughout the world found higher reported rates of anxiety, depression, post-traumatic stress disorder (PTSD), psychological distress and stress during the pandemic, than in pre-pandemic times [15].

Within disaster MH research, several factors have been found to influence MH, particularly post-traumatic stress symptoms (PTSS). A conceptual model of post-disaster functioning incorporates demographic characteristics, traumatic exposure to the disaster (e.g., threats or loss), and aspects of the recovery context (e.g., ability to cope, perceived stress or life stressors, perceived social support) [16]. This model accounted for 62% of the variance in PTSS in past research by including demographics, disaster exposure, social support, and coping [17]. Perceived social support has been linked to resilient outcomes after disasters, such as the 9/11 terrorist attack in New York City and the SARS bio-epidemic, even after controlling for demographic and other predictor variables [14,18]. Research is needed to add to the existing documentation of COVID-19 impacts on GSWs’ MH, while incorporating these factors.

The current study fills an important gap in understanding the impact of demographics, disaster fear and threat (COVID-19 fear and workplace threat perception), perceived stress, and social support (lack of from family and friends) on GSWs’ MH outcomes (anxiety, depression, and PTSS), drawing from a disaster MH conceptual model [16]. This study also qualitatively explores what supports GSWs report needing to carry out their jobs, which add context and depth to the quantitative measures.

## 2. Materials and Methods

### 2.1. Procedure

The research team formed a partnership with a large Californian union to develop and disseminate a survey to active GSW union members. The union represents approximately 30,000 GSWs at various Californian grocer retailers. A group of administrative union staff and representatives and the study authors finalized a survey to disseminate in July of 2020. Researchers obtained institutional review board (IRB) approval from a major university and collected data through an online Qualtrics questionnaire in English and Spanish. Data collection began in November and ended in early January of 2021. Union members were recruited by the union through email and text invitations sent about once per week. A total of 24,639 GSWs had successfully received the invitation to complete the survey. Participants had the choice to opt in for a raffle to win one of (38) $20.00 grocery store e-gift cards. They also indicated if they consented to being contacted for possible follow-up surveys and provided contact information.

### 2.2. Measures

#### 2.2.1. COVID-19 Fear

The Fear of COVID Scale (FCV-19S) consists of seven items assessing the degree to which a participant is afraid of COVID-19 on a five-point Likert scale [19]. In the current study, participants rated their level of agreement with statements about COVID-19 on a four-point Likert scale, ranging from *strongly disagree* (1) to *strongly agree* (4). Sample items include: “It makes me uncomfortable to think of COVID-19”, “I cannot sleep because I am worrying about COVID-19”, etc. Scores for the items were summed, ranging from a total of seven to 28. The scale demonstrated good reliability and internal validity and has been translated into several languages and used around the world [20]. In the current sample, Cronbach’s α = 0.9.

#### 2.2.2. Workplace Threat Perception

This scale was created specifically for this study to assess possible threat perception and anxiety about following safety guidelines (e.g., enforcing mask-wearing policies, speaking up about health and safety concerns) in the workplace. With input from union leaders and representatives, a total of seven items were created. Sample items include: “I have felt like I was in danger for enforcing a mask policy at my store”, “I worry about being retaliated against if I speak up about health and safety concerns”, “I worry that I will be victimized for following the rules”, etc. Respondents rated the extent to which they agreed with these statements on four-point Likert scale, ranging from *strongly disagree* (1) to *strongly agree* (4). The items were summed into a total score, ranging from seven to 28. For this sample, Cronbach’s α = 0.9.

#### 2.2.3. Perceived Stress

The Perceived Stress Scale (PSS-10) is a 10-item scale assessing the degree to which participants perceive general situations in their lives as unpredictable, uncontrollable, or overwhelming during the past month [21]. Sample items include: “How often have you…” “found that you could not cope with all the things you had to do,” “been able to control irritations in your life,” “felt that things were going your way?” Response options used a five-point Likert scale and range from *never* (0) to *very often* (4). Items were summed to create a total score. In this sample, Cronbach’s α = 0.87.

#### 2.2.4. Social Support

The Perceived Social Support Scale (PSSS) is a 12-item scale measuring perceived social support received from friends, family, and significant others [22]. For this study, only the two four-item subscales for friends and family were used. Respondents were asked to rate how about much they agreed with statements about the level of social support from their family (“I can talk about my problems with my family”) and friends (“I can count on my friends when things go wrong”). Response options ranged from *strongly disagree* (1) to *strongly agree* (5) on a 5-point Likert-type scale. Each item was reverse coded to represent a lack of social support. Items were then summed to create a total score for the family and friend subscales. In the current sample, Cronbach’s α = 0.91 and 0.90, respectively for each subscale.

#### 2.2.5. Generalized Anxiety Symptoms

The two-item Generalized Anxiety Disorder scale (GAD-2) was used to measure self-reported anxiety symptoms [23]. The stem for the items was, “Over the last two weeks, how often have you been bothered by the following problems”, and a sample item was: “worrying too much about different things”. The response choices are as follows: *not at all* (0), *several days* (1), *more than half the days* (2), and *nearly every day* (3). Items were summed to create a total score. The present sample, Cronbach’s α = 0.86.

#### 2.2.6. Depressive Symptoms

The Patient Health Questionnaire-9 (PHQ-9) is a nine-item self-report measure of symptoms of depression [24]. Participants were asked how often they have experienced depressive symptoms within the last two weeks (e.g., “feeling down, depressed, or hopeless”) and response options are as follows: *not at all* (0), *several days* (1), *more than half the days* (2), and *nearly every day* (3). Scores were summed to create a total score. In the current sample, Cronbach’s α = 0.9.

#### 2.2.7. Post-Traumatic Stress Symptoms (PTSS)

PTSS were assessed using the Impact of Events Scale-6 (IES-6), which is a 6-item measure derived from the 22-item Impact of Events Scale-Revised (IES-R) [25]. Participants were asked to rate how distressed or bothered they were regarding their experiences pertaining to the COVID-19 pandemic within the last seven days. Response options are as follows: *not at all* (0), *a little bit* (1), *moderately* (2), *quite a bit* (3), and *extremely* (4), and were summed together to create a total score. Two items each assessed intrusion (e.g., “I thought about it when I didn’t mean to”), avoidance (e.g., “I tried not to think about it”), and hyperarousal (e.g., “I felt watchful or on guard”). The IES-6 proved to be highly correlated with the IES-R and is considered a strong, brief measure of posttraumatic stress reactions in clinical and nonclinical populations [26]. A sum score was calculated. Cronbach’s α = 0.88 in the current sample.

#### 2.2.8. Recommendations to Improve GSWs’ Ability to Work

Respondents were asked an open-ended question: “*What support are you needing right now to be able to carry out your work?*” to gather information about possible needs or recommendations for support. No limit was placed on the open-ended question and respondents wrote as little or as much as they wanted.

#### 2.2.9. Demographics

Participants were asked their sex, gender identity, ethnicity, and age (see Table 1). They also provided information about their work position, average number of hours worked per week, and length of time working in the grocery store industry.

### 2.3. Analytic Plan

First, an a-priori analysis was conducted using a popular tool, GPower 3.1 (Heinrich Heine University Düsseldorf, Düsseldorf, Germany) to determine the adequate sample target size which was obtained in this current study [27,28]. SPSS Version 27 (International Business Machines [IBM] Corporation, Armonk, NY, USA) was used for all subsequent analyses. Variables were assessed for normality and Pearson correlations were conducted to assess the strength and direction of the bivariate relationships among the variables. Next, differences by gender identity among study variables were assessed using an independent samples *t*-test. Differences by age and ethnicity were each assessed using a one-way analysis of variance (ANOVA). Post-hoc Tukey HSD tests revealed differences, which provided rationale for including age and gender identity as covariates. Lastly, three hierarchical linear regression models were conducted to examine the variance in MH outcomes (i.e., depression, anxiety, and PTSS symptoms,) accounted for by demographic variables (i.e., age and gender identity), disaster fear and threat (i.e., COVID-19 fear and workplace threat perception), perceived stress, and social support (lack of from family and friends).

Qualitative responses for the open-ended question, “*What support are you needing right now to be able to carry out your work*?” were analyzed for themes and informed by thematic analysis guidelines [29]. Initial codes were used based on the survey question (what supports are needed) and refined after inductive codes were identified. Two authors independently coded all data and came to a consensus on codes and themes through the discussion.

## 3. Results

### 3.1. Sample Characteristics

The final sample included 842 participants who self-identified as GSWs, which equaled a 3.4% response rate. Over half of the sample identified their gender identity as female (62.1%), 36.2% as male, and the remaining as non-binary or other (1.6%). Their ages ranged from 18–69 years old (*M* = 41.5, *SD* = 13.9). Additionally, the sample was diverse and worked in a range of GSW positions (see Table 1). Almost a quarter of respondents (24.5%) had worked in the industry for 20+ years and the majority (62%) reported working an average of 20–40 h.

### 3.2. Preliminary Analyses

The variables were examined to ensure that multicollinearity did not exist, or only at very low levels [30]. The Durbin–Watson test for independence of residuals was also utilized; results indicated the values of the residuals were independent [31]. Analyzing plots of standardized residuals to evaluate for assumptions of normality, homoscedasticity, linearity, independence of errors, and absence of outliers indicated that the data was free of any violations of assumptions [30].

#### 3.2.1. Correlations

All variables were significantly and positively related to depression, anxiety and PTSS as expected (see Table 2).

#### 3.2.2. Differences by Ethnicity, Gender Identity, and Age

Ethnicity. For ethnicity, significant differences were found only for COVID-19 fear. Latino participants were significantly more fearful of COVID-19 (*M* = 17.28, *SD* = 5.24) than White participants (*M* = 15.08, *SD* = 5.17; *p* < 0.001).

Gender Identity. For gender identity, females were found to report significantly higher levels of COVID-19 fear, perceived stress, anxiety, depression, and PTSS than males (see Table 3). No other differences were found by gender identity.

Age. Significant differences were found by age for COVID-19 fear, workplace threat perception, perceived stress, lack of familial support, and MH outcomes (see Table 4). The 18–29-year-old age group reported significantly greater workplace threat perception than the 50-59 (*p* = 0.01) and 60–69-year-old age groups (*p* = 0.05). They also reported significantly higher perceived stress (*p* = 0.001) than the 40–49-year-old group (*p* = 0.001), 50–59-year-old group (*p* < 0.001) and 60–69-year-old group (*p* < 0.001). For perceived stress, the 30–39-year-old group also reported significantly more stress than 60–69-year-old age range (*p* = 0.04). In terms of social support, the 18–29-year-old age group reported a significantly greater lack of familial social support than the 40–49 (*p* = 0.01) and 60–69-year-old age groups (*p* = 0.02).

Regarding MH outcomes, the 18–29-year-old age group significantly reported greater anxiety and PTSS than the 50–59 (*p* = 0.002) and 60–69-year-old age groups (*p* = 0.002). For the 30–39-year-old age group, they also reported significantly higher PTSS than the 50–59 (*p* = 0.05) and 60–69-year-old age groups (*p* = 0.03). Additionally, the 18–29-year-old age group reported significantly greater depression scores than all age groups (30–39, *p* = 0.05; 40–49, *p* = 0.02; 50–59, *p* < 0.001; 60–69, *p* = 0.02) No differences were found in COVID-19 fear and lack of social support from friends by age.

### 3.3. Hierarchical Multiple Regression Models Predicting MH

#### 3.3.1. Hierarchical Multiple Regression Predicting Anxiety

The hierarchical multiple regression revealed that at Step one, COVID-19 fear, and workplace threat perception experiences contributed significantly to the regression model and accounted for 27.4% of the variation in anxiety symptoms (see Table 5). Introducing the perceived stress variable explained an additional 20.9% of variation in anxiety and this change in *R*^2^ was also significant. Adding lack of social support to the regression model did not explain additional variation in anxiety, yet this change in *R*^2^ was significant.

When all five predictor variables were included in stage three of the regression model, only COVID-19 fear and perceived stress were significant predictors of anxiety and accounted for 48.2% of the variance in anxiety.

#### 3.3.2. Hierarchical Multiple Regression Predicting Depression

At Step one, COVID-19 fear, and workplace threat perception contributed significantly to the regression model and accounted for 24.5% of the variation in depression (see Table 5). Introducing perceived stress explained an additional 19.5% of variation in depression, which was also significant. Adding lack of social support from family and friends to the regression model did not explain additional variance in depression, yet this change in *R*^2^ was significant. Only COVID-19 fear, workplace threat perception, and perceived stress predicted depression. Altogether, all variables in the model accounted for 44.6% of the variance in depression.

#### 3.3.3. Hierarchical Multiple Regression Predicting PTSS

The hierarchical multiple regression model predicting depression had similar results to the model predicting PTSS. The final hierarchical multiple regression revealed COVID-19 fear, and workplace threat perception contributed significantly to the regression model and accounted for 36% of the variation in PTSS (see Table 5). Introducing perceived stress explained an additional 5.8% of variation in PTSS and this change in *R*^2^ was also significant. Adding social support to the regression model did not explain further variation in PTSS; however, this change in *R*^2^ was significant. COVID-19 fear, workplace threat perception, and perceived stress were predictive of PTSS, which explained 41.7% of the variance in PTSS.

### 3.4. Assessing Psychosocial Predictors of Mental Health Outcomes

#### 3.4.1. Step 1: Disaster Fear and Threat

As shown in Table 5, COVID-19 fear was significantly and positively related to anxiety, while COVID-19 fear and workplace threat perception were positively related to depression and PTSS. COVID-19 fear accounted for 27.4% of the variance in anxiety, and COVID-19 fear and workplace threat perception accounted for 24.5% of the variance in depression, and 36% in PTSS.

#### 3.4.2. Step 2: Perceived Stress

COVID-19 fear and perceived stress were significantly and positively related to anxiety (see Table 5). COVID-19 fear and workplace threat perception, in addition to perceived stress, were significantly and positively related to depression and PTSS. Adding perceived stress to the model accounted for 48.3% of the variance in anxiety, 44% in depression, and 41.8% in PTSS.

#### 3.4.3. Step 3: Social Support

COVID-19 fear and perceived stress were significantly and positively related to anxiety, while lack of familial social support and social support from friends were not (see Table 5). For the other models, COVID-19 fear, workplace threat perception, and perceived stress were significantly and positively related to depression and PTSS, while the social support variables were not. No changes in the previous variables occurred once lack of social support was added into the model. Lack of social support from family and friends did not account for an increase in variance for anxiety (48.2%) nor PTSS (41.7%), and accounted for a negligible increase in depression (44.6%).

### 3.5. Qualitative Themes of Recommendations

Table A1 in Appendix A shows groupings of various recommendations made by a subset of GSWs (*n* = 458). A total of 557 unique suggestions were made (GSWs could provide more than one) and were broken down into the following five qualitative themes: enforcement and implementation of protection policies for worker safety (e.g., store capacity limits, storewide adherence to public health guidelines, mask mandates), institutional and leadership support (e.g., from corporate, management, and union leaders), work accommodations and compensation (e.g., hazard pay, paid time off due to COVID-19, flexibility when sick), access to PPE and hygiene supplies, and MH and emotional support (e.g., appreciation, encouragement, MH services, therapy). Less than a quarter of participants (17%) indicated that they did not have any recommendations to provide.

## 4. Discussion

GSWs have worked on the frontlines since the start of the COVID-19 pandemic, and little is understood about how experiences of fear, threat, perceived stress, and social support impact their MH. Based on a theoretical framework of disaster MH, three hierarchical linear regression models were run to predict MH outcomes. The results revealed that COVID-19 fear, workplace threat perception, and perceived stress explained almost half of the variation for depression and PTSS symptoms, while COVID-19 fear and perceived stress explained almost half of the variance for anxiety symptoms. Within the anxiety model, workplace threat perception was approaching significance. The results confirm previous research, which found that reports of feeling unsafe in the workplace was the most robust predictor of MH of GSWs in Arizona [10]. As evidenced by the results, a variety of MH reactions may be possible during this ongoing pandemic, and higher perceptions of fear or threat and higher perceived stress levels may contribute to them.

Contrary to previous disaster research, a lack of social support was not predictive of MH outcomes [17]. Social support has been well-documented as a robust predictor of post-disaster resilient outcomes [14]. Our findings are inconsistent with previous findings and warrant further investigation. The quality or types of perceived social support may differ from other disasters due to the unique nature of COVID-19 and the restrictions that were placed on social gatherings. It is largely unclear from our study whether participants’ social support changed due to COVID-19. We also do not know the forms of social support that participants had received (e.g., in-person, over video chat, phone call, text message). Most studies have also focused on the impact of social support post-disaster, not during. Further research is needed to understand the underlying mechanisms of social support as it relates to the current pandemic, especially for GSWs.

The findings of the study also revealed that workplace threat perception, perceived stress, lack of family support, and MH outcomes were found to be heightened for younger GSWS, ages 18–29. Older adults have been found to have less psychological distress than young adults during the COVID-19 pandemic in other studies [32]. Other disaster research suggests older adults may be buffered against stress [33,34]. In the current study, comparisons revealed no differences in COVID-19 fear among age groups, even though older adults (starting at age 50 and above) are at greater risk of adverse outcomes [35]. More research is needed to understand the impact of COVID-19 on younger versus older GSWs and adults in general.

Overall, the model used within the current study can serve as an initial guide to understanding MH outcomes during the pandemic. Studying MH during the pandemic is challenging because we are not in a post-disaster context, and the crisis is continually evolving. GSWs may continue to be exposed to COVID-19 and impacted by it as time goes on, and so MH outcomes must be continually assessed. Efforts to conceptualize and measure experiences relating to COVID-19 (e.g., loss of loved ones to the virus), in addition to perceptions of fear and threat, should be made.

The qualitative responses by GSWs also possessed rich information about the varying supports that are needed for them to effectively carry out their work. Work conditions are integral to physical and MH, and overall well-being [2]. The participants’ recommendations mainly related directly to the workplace environment (e.g., enforcement and implementation of protection polices for worker safety, institutional and leadership support, work accommodations and compensation, access to PPE). These suggestions were very much aligned with recommendations made from a review of workplace factors contributing to COVID-19 related risks and physical and MH impacts [2]. Employers possess an ethical and moral duty to protect GSWs and should strive to uphold safe and comfortable working conditions that reduce or eliminate identified stressors or hazards [2]. From a social justice perspective, securing a safe workplace environment is integral to GSWs’ well-being and rights, and may assist in reducing the perpetuation of existing social and health inequalities for this vulnerable group [2]. Even without local and federal regulations, employers can take action to protect GSWs through efforts to put forth strong occupational health and safety policy measures or legislation. Such efforts may help to improve working conditions on a large-scale level [2].

Employers, upper management, or unions of GSWs can work together to implement additional measures and supports in the workplace (e.g., greater protections within store, such as mask wearing mandates by individuals other than GSWs, limiting the number of individuals within store, increased hazard pay, MH services, providing information about how to access vaccines). Employers may consider engaging in a number of actions to support their workers such as establishing strong occupational health and safety programs in line with public health guidelines, assessing risk in the workplace, allowing employees to express concerns through surveys, interviews, or informal meetings without fear of later consequences for speaking up, identifying employees at-risk of adverse physical or MH outcomes, providing education on how employees can protect themselves, allowing GSWs greater flexibility and paid time off for COVID-19 related issues (without fear of losing their job), and disseminating information about physical and MH services or resources to assist employees in managing distress, health, or psychological issues [2]. Engaging in such acts may build trust among employers and GSWs, increase GSWs’ feelings of safety, care, and appreciation, build resiliency [2], and reduce the risk of experiencing moral injury, distress, and long-term MH consequences. It would also be beneficial for employers, management, or union leaders to closely document implemented safety measures and assess the potential impacts they have on GSWs to determine if they are associated with reductions in fear, workplace threat perceptions, and MH outcomes.

GSWs may also benefit from stress-reduction strategies and MH treatment which may lessen fear, perceived stress, and MH symptoms associated with work and the impacts of the COVID-19 pandemic. MH support can be provided in the form of counseling or psychotherapy from a trained MH professional. GSWs can learn coping mechanisms and strategies to reduce MH symptoms and stress; however, barriers to accessing MH treatment may exist, or there may be a lack of knowledge about how to initiate or seek out services. Apart from therapy, GSWs can also employ other stress-reduction strategies such as engaging in physical exercise [36], spiritual, religious, or mindfulness and meditation practices [37,38], or eating a healthy diet [39], which have shown to be effective against reducing stress and stress-related outcomes. GSW leaders or unions can assist GSWs in understanding how to access MH services or provide information regarding MH treatment in general, or other stress-reduction suggestions.

Additionally, MH professionals can play an important role in supporting the MH of GSWs and other essential workers. Some MH providers have joined together to expand their services to provide MH support to health care colleagues in the form of volunteer work (e.g., in Washington state) [40]. Networks of MH professionals providing low cost or free services to essential workers could compiled and their information shared and disseminated to GSWs, in addition to health care and other workers. MH professionals can also engage in advocacy work illuminating the need for protection, access to MH services, and support for GSWS and other essential workers throughout the world [41]. The World Health Organization (WHO) has identified several strategies to guide the strengthening of MH care and accessibility, which include working to reform MH care systems and investing in MH workers [41]. Additionally, MH providers may need to be aware of the unique psychosocial influences of the COVID-19 pandemic on GSWs. Their experiences during the pandemic may differ from those of health care workers (e.g., in terms of implemented protections, status, public perception, and treatment), and other essential workers. MH treatment modalities and psychosocial tools can also be tailored for use within the COVID-19 context [41]. MH providers may be treating long-term MH consequences of the COVID-19 pandemic for years to come and should keep up to date with emerging and changing developments in the medical and psychological fields [42].

Given the graveness of the COVID-19 pandemic and its impact on GSWs, it is critical to continue to examine predictors of MH, particularly those specifically relating to workplace conditions and safety, and MH outcomes themselves. This study provides a snapshot into MH outcomes at a particular time during the COVID-19 pandemic, thus there are limitations to what can be inferred long-term. Rates of distress and MH symptom levels are higher during the pandemic, than pre-pandemic, even in general populations without previous MH conditions [43]. Despite this, past research has found most individuals to be resilient after disaster [14]. Continued research is needed to increase our understanding of the impact of the workplace environment and the COVID-19 pandemic on GSWs. Because GSWs are crucial to society and provide critical infrastructure, it is important for employers and management to serve as advocates for their safety and protection within the workplace, which may affect their MH.

### 4.1. Limitations

Certain limitations should be acknowledged. First, since most respondents were solicited via an email/text list through a union in California, the findings may not generalize to all GSWs or those belonging to unions with varied levels of support. Additionally, the response rate for the current study was very low in comparison to the number of individuals who had received the survey invitation, thus there is a selection bias. Only those participants who were interested in the survey replied and may have unique characteristics, which may not be generalizable to the general GSW population. Thus, the results of the study should be interpreted with caution. The cross-sectional design of this study also makes it difficult to assess temporal priority among variables. Measuring independent and dependent variables from a single respondent can increase risk of shared method variance, which can inflate associations. We also lack information about previous MH history or functioning and experiences of other traumatic events, which are also known to be predictive of post-disaster adjustment and resiliency [14,44]. Lastly, focus groups or interviews would have provided a richer depth of knowledge about GSWs’ experiences, support needed, and MH.

### 4.2. Future Considerations

Detailed aspects of essential workers’ work environments should be measured and assessed to better understand feelings of fear, threat and safety, and their relation to MH and well-being. More robust mixed-methods or qualitative studies should be implemented to expand understanding. It is also necessary to employ longitudinal methodological approaches to examine associations over time. COVID-19 is a chronic, everchanging disaster, thus, long-term outcomes are unknown. It is also difficult to tease out the influence of compound stressors in the GSW population. This could be better delineated with a comparison to another sample of other essential workers, such as medical workers, nurses, teachers, or others.

### 4.3. Implications

GSWs may be a vulnerable group at-risk of contracting COVID-19 and experiencing heightened levels of fear, threat, and stress, especially those aged 18–29-years-old. Grocery store management and unions must do everything in their ability to increase protections in the workplace, as this may reduce perceptions of fear, threat, and stress, which may be associated with fewer MH symptoms. Some recommendations include repeat and routine COVID-19 employee testing, increased enforcement of CDC safety guidelines within store, hazard pay, comprehensive employee assistance or MH referrals to help GSWs cope with psychological distress, increased displays or expressions of appreciation and care from customers and management, and creation of social networks (e.g., open forums, support groups) for GSWs to connect with each other to provide and receive social support.

## 5. Conclusions

COVID-19 fear and perceived stress were predictive of anxiety within GSWs, while COVID-19 fear, workplace threat perception, and perceived stress were predictive of depression and PTSS. Social support and demographic characteristics were not found to be predictive of MH outcomes. Feelings of fear, threat, or safety, particularly in the workplace, and the ability to cope with stress, are important factors when considering GSWs’ MH symptoms. Increased protections, policies, and supports should be implemented within the workplace, which may improve GSWs’ feelings of safety and reduce overall MH impacts.

## Figures and Tables

**Table 1:** Participant demographic and work characteristics (*n* = 842).

Characteristic	*n*	%
Ethnicity		
Asian	34	4
African American	28	3.3
Native American	6	0.7
Native Hawaiian or Pacific Islander	4	0.5
Latino	280	33.3
White	222	72.2
Other	34	4
Declined to State	234	27.8
GSW Position		
Baker	24	2.9
Butcher	16	1.9
Cashier	202	24
Courtesy Clerk	116	13.8
Department Manager	81	9.6
Florist	11	1.3
Janitorial	1	0.1
Service Deli Worker	58	6.9
Stocker	75	8.9
Store Manager	6	0.7
Other	242	28.7
Declined to state	10	1.2
Length of Time Worked in Years		
0–1	184	22
1–5	205	24.3
5–10	101	12.1
10–20	141	16.7
20+	206	24.5
Average Number of Hours Worked Per Week		
0–10	9	1.1
10–20	69	8.2
20–40	522	62
40–60	209	24.8
60+ hours	3	0.4

Note. GSW = grocery store worker.

**Table 2:** Means, standard deviations, and bivariate correlations (*n* = 842).

Variables	1	2	3	4	5	6	7	8	9	10
1. Age	1									
2. GE	−0.12 **	1								
3. FEAR	−0.06	−0.18 ***	1							
4. WPT	−0.15 ***	0.03	0.39 ***	1						
5. PS	−0.25 ***	−0.09 *	0.38 ***	0.39 ***	1					
6. FAM	−0.12 **	0.05	0.02	0.11 **	0.16 **	1				
7. FRND	−0.03	0.01	0.15 ***	0.10 *	0.11 **	0.59 ***	1			
8. ANX	−0.19 ***	−0.10 *	0.44 ***	0.35 ***	0.66 **	0.12 **	0.09 *	1		
9. DEP	−0.21 ***	−0.07	0.38 ***	0.35 ***	0.66 **	0.21 ***	0.17 ***	0.70 ***	1	
10. PTSS	−0.17 ***	−0.12 **	0.53 ***	0.41 ***	0.53 **	0.10 **	0.10 *	0.60 ***	0.64 ***	1
*M*	41.56	-	16.23	16.85	18.38	8.02	8.18	2.36	8.19	9.70
*SD*	13.91	-	5.38	6.71	7.18	4.41	4.06	1.93	6.60	6.00

Note. GEN = gender identity (male or female; female = 1); FEAR = COVID-19 fear; WPT = workplace threat perception; PS = perceived stress; FAM = lack of familial support; FRND = lack of support from friends; ANX = anxiety; DEP = depression; PTSS = post-traumatic stress symptoms. * *p* < 0.05, ** *p* < 0.01, *** *p* < 0.001.

**Table 3:** Differences among variables of interest by gender identity (*n* = 600).

Variables	Females (*n* = 379) M (SD)	Males (*n* = 221) M (SD)	*t*-Value	Df	*p*-Value
COVID-19 Fear	17.11 (5.33)	14.82 (5.10)	5.22	474.35	<0.001
WPT	16.82 (6.67)	16.96 (6.77)	−0.24	595	0.814
Perceived Stress	19.21 (6.89)	17.18 (7.37)	3.34	435.60	<0.001
Lack of SS from Family	7.93 (4.43)	8.00 (4.16)	−0.19	596	0.851
Lack of SS from Friends	8.14 (4.03)	8.21 (3.92)	−0.22	594	0.414
Anxiety	2.56 (1.94)	2.00 (1.78)	3.60	492.32	<0.001
Depression	8.75 (6.51)	7.14 (6.33)	3.00	468.51	0.002
PTSS	10.46 (5.97)	8.59 (5.65)	3.83	478.63	<0.001

Note. WPT = workplace threat perception; SS = social support.

**Table 4:** One-way Analyses of Variance (ANOVA) in variables of interest by age group (*n* = 590).

Variables	Age Group					ANOVA Results		
	18–29 M (SD) (*n* = 146)	30–39 M (SD) (*n* = 109)	40–49 M (SD) (*n* = 126)	50–59 M (SD) (*n* = 159)	60–69 M (SD) (*n* = 50)	df	F	η^2^
FEAR	16.14 (5.34)	16.97 (5.90)	17.21 (5.73)	15.79 (4.87)	14.86 (4.39)	588	2.64 *	0.04
WPT	18.36 (6.78)	17.58 (6.42)	17.13 (6.67)	15.87 (6.73)	15.38 (6.55)	586	3.62 **	0.05
PS	21.41 (7.40)	19.32 (6.61)	18.08 (6.98)	17.18 (6.46)	15.92 (7.65)	588	9.91 ***	0.10
FAM	9.07 (4.42)	8.01 (4.43)	7.39 (4.30)	7.99 (4.51)	6.86 (3.48)	588	3.66 **	0.05
FRND	8.08 (4.16)	8.50 (4.23)	8.37 (4.04)	8.28 (3.96)	7.24 (3.38)	586	0.96	0.02
ANX	2.86 (1.84)	2.53 (1.91)	2.56 (1.95)	2.05 (1.85)	1.71 (1.80)	587	5.57 ***	0.07
DEP	10.71 (7.28)	8.47 (6.54)	8.33 (6.07)	7.00 (6.10)	6.78 (5.68)	587	7.30 ***	0.08
PTSS	10.85 (6.01)	10.74 (6.12)	10.33 (6.29)	8.73 (5.06)	7.82 (60.8)	586	4.83 ***	0.06

Note. FEAR = COVID-19 fear; WPT = workplace threat perception; PS = perceived stress; FAM = lack of familial support, FRND = lack of support from friends; ANX = anxiety; DEP = depression, PTSS = post-traumatic stress symptoms. * *p* < 0.05, ** *p* < 0.01, *** *p* < 0.001.

**Table 5:** Summary of hierarchical linear regression models predicting MH outcomes.

	Anxiety (*n* = 565)			Depression (*n* = 566)			PTSS (*n* = 558)		
Predictor	ß	F(df)	R^2^	ß	F(df)	R^2^	ß	F(df)	R^2^
Covariates:		16.15(2) ***	0.051		16.09(2) ***	0.051		13.58(2) ***	0.043
Age	−0.19 ***			−0.21 ***			−0.16 ***		
GEN	0.15 ***			0.12 **			0.15 **		
Step 1:		54.20(4) ***	0.274		47.00(4) ***	0.245		80.46(4) ***	0.360
Age	−0.14 ***			−0.16 ***			−0.11 **		
GEN	0.08 *			0.07			0.06		
FEAR	0.36 ***			0.28 ***			0.44 ***		
WPT	0.21 ***			0.26 ***			0.24 ***		
Step 2:		106.51(5) ***	0.483		89.85(5) ***	0.440		82.10(5) ***	0.418
Age	−0.04			−0.06			−0.06		
GEN	0.03			0.02			0.03		
FEAR	0.23 ***			0.15 ***			0.37 ***		
WPT	0.06			0.12 ***			0.16 ***		
PS	0.53 ***			0.51 ***			0.28 ***		
Step 3:		76.05(8) ***	0.482		66.17(7) ***	0.446		58.80(7) ***	0.417
Age	−0.04			−0.05			−0.05		
GEN	−0.03			0.02			0.03		
FEAR	0.24 ***			0.16 ***			0.38 ***		
WPT	0.06			0.11 **			0.15 ***		
PS	0.53 ***			0.50 ***			0.28 ***		
FAM	0.02			0.07			0.05		
FRND	−0.04			0.03			−0.03		

Note. GEN = gender identity (male or female; female = 1); FEAR = COVID-19 fear; WPT = workplace threat perception; PS = perceived stress; FAM = lack of familial support, FRND = lack of support from friends. * *p* < 0.05, ** *p* < 0.01, *** *p* < 0.001.

## Data Availability

Data was made available to the public and can be accessed at the following link: https://doi.org/10.6084/m9.figshare.14970012.v1 (accessed on 12 July 2021).

## Appendix A

A supplemental table is provided within the Appendix. Table A1, titled *Thematic Coding of Suggestions Made by GSWs to Improve Their Ability to Work* (*n* = 458), contains groupings of various recommendations and supports needed in the workplace made by a subset of GSWs, along with examples of specific quotes.

**Table A1:** Thematic coding of suggestions made by GSWs to improve their ability to work (*n* = 458).

Thematic Themes	*n*	%	Direct Quotations from Participants
Enforcement and Implementation of Protection Policies for Worker Safety	134	24	*“I would like stronger enforcement of mask wearing for customers or even more security for unreasonable customers and easier access to covid tests…”* *“I just need someone there [to enforce policy], or the proper policies in place to help us stay clean and safe in the workplace…”* *“Enforce capacity limits.”*
Institutional and Leadership Support	106	19	*“Upper management needs to be more proactive and supportive and currently strongly lacks leadership skills with regards to COVID-19 preparation and implementing CDC guidelines in the grocery industry.”* *“I need the company to care more about people, their workers, and our health rather than making more and more money. It’s never enough.”* *“More union people checking-in to see the conditions at work.”*
Work Accommodations and Compensation	95	17	*“…more compensation, I’m already struggling financially and walking this tightrope simply adds to the stress of the state of things.”* *“We need to be getting hazard pay for being at a high risk every day. We don’t get paid enough for all that we do. We are around hundreds of people a day…”* *“I wish I could take time off and be compensated. I worry I will bring COVID-19 home to my family and now my co-workers are all testing positive.”*
PPE and Hygiene Supplies	88	16	*“More PPE. More sneeze guards in all open windows. More gloves, cleaning supplies, face shields, Lysol spray…”* *“Making sure the workers are protected and supplied with proper PPE (i.e., correct size gloves, masks that protect).”*
Mental Health and Emotional Support	38	7	*“We need to know that our efforts are being appreciated and we are valued as essential workers.”* *“I need the support of family and coworkers as it gets harder and harder for me to show up to work. I feel overworked and unappreciated.”* *“Essential workers need free counseling or therapy due to these difficult times!”*
No Recommendations	96	17	*“None, my workplace has done enough.”*
Total	557	100	

Note. Respondents (*n* = 458) could provide more than one endorsement, hence a total of 557 separate suggestions.
